# Isolated Cecal Necrosis as a Cause of Acute Abdomen

**DOI:** 10.3390/jcm14031019

**Published:** 2025-02-05

**Authors:** Oguzhan Sunamak, Kadir Corbaci, Cebrail Akyuz, Mehmet Onur Gul, Evren Besler, Turgut Donmez, Feza Ekiz

**Affiliations:** 1 Department of General Surgery, University of Health Sciences, Haydarpasa Numune Training and Research Hospital, 34668 Istanbul, Turkey; 2 Mustafa Selahattin Çetintaş State Hospital, 11500 Bilecik, Turkey; 3 Department of General Surgery, University of Health Sciences, Gaziantep City Hospital, 27470 Gaziantep, Turkey; 4 Department of General Surgery, University of Health Sciences, Bakirkoy Sadi Konuk Training and Research Hospital, 34147 Istanbul, Turkey; 5 Department of General Surgery, Istanbul Medical Faculty, University of Istanbul, 34093 Istanbul, Turkey

**Keywords:** cecum, necrosis, colectomy, end-ileostomy, anastomosis, emergency

## Abstract

**Background/Objectives:** Isolated cecum necrosis (ICN) is a rare disease and there are few reports on it in the literature. We retrospectively analyzed the patients with acute abdomen who had ICN, in terms of etiology, demographic findings, surgery, and prognosis to determine the best treatment approach. **Methods:** Seven patients with per-operative diagnosis of isolated cecal necrosis were included in the study. Age, gender, comorbidities, symptoms, laboratory data, imaging modalities, time from symptoms to surgery, surgical procedure, hospital stay, morbidity, and mortality were retrospectively analyzed. **Results:** The median age of the patients and the F/M ratio were 61 years and 2/5, respectively. The most common comorbidity was chronic renal failure, followed by hypertension, diabetes mellitus, and coronary artery disease. The median time from symptom onset to surgery was 1 day. The ileocolic anastomosis was performed in 4 patients and terminal ileostomy in three patients. The morbidity and mortality rates were 28.5% and 14.3%, respectively. The median length of hospital stay was 12 days. **Conclusions:** ICN should be suspected in elderly patients with comorbidities presenting to the emergency department with an acute abdomen. Computed tomography is an important diagnostic tool. Diagnostic laparoscopy is a good option for definitive diagnosis and end ileostomy is better than anastomosis in patients with bleeding tendency or hypotensive patients with chronic renal failure on dialysis, coronary artery disease, and in the presence of purulent peritonitis to reduce morbidity and mortality.

## 1. Introduction

Isolated cecal necrosis (ICN) is a rare cause of acute abdomen, especially in elderly patients with comorbidities. However, ICN can also occur without any underlying cause [1,2,3].

Colonic ischemia generally results from occlusive or non-occlusive causes. Occlusive ischemia results from reduced blood flow in arterial thromboembolism or venous thrombosis and non-occlusive factors in posttraumatic situations, chronic hemodialysis patients, after open heart surgery, or digoxin use [1,2,3,4]. It may also occur as a complication of Behcet’s disease [5].

ICN causes right lower quadrant pain and can be confused with many conditions causing right lower quadrant tenderness, especially acute appendicitis [2,3,4,5,6]. Sometimes, it may present as a colonic mass and be confused with a malignancy [7,8].

However, because it is rare, it is usually not considered in the differential diagnosis of right lower quadrant pain. Early diagnosis and surgical treatment of patients with isolated cecal necrosis is critical because of the risk of cecal perforation and sepsis [2]. The majority of patients are operated on with a prediagnosis of acute appendicitis, using a McBurney incision, and a second incision may be indicated.

We retrospectively analyzed the patients who underwent surgery for acute abdomen at SBU Haydarpasa Numune Training and Research Hospital between 2010 and 2021 and were diagnosed with isolated cecal necrosis in terms of etiology, demographic findings, surgery and prognosis to determine the correct approach to these patients for diagnosis and treatment.

## 2. Materials and Methods

Seven patients who underwent surgery in a tertiary hospital emergency department between 2010 and 2021 and whose pathology revealed cecal necrosis were included in the study. All patients provided informed consent. The study also received institutional review board approval for retrospective studies (approval number: E62977267-771; 30 March 2021).

Age, sex, comorbidities, symptoms, laboratory data, imaging reports, symptom-to-surgery interval, surgical procedure, hospital stay, morbidity, and mortality were retrospectively analyzed.

## 3. Results

Table 1 shows patient demographics. Patients with and without perforation were divided into two groups, and the results were compared (Table 2).

The median age of the patients was 61 (range 36–67) years. The F/M ratio of the patients was 2/5. Renal failure (CRF) was present in four (57.1%), hypertension (HT) in two (28.6%), diabetes mellitus (DM) in two (28.6%), coronary artery disease (CAD) in two (28.6%), and a history of cerebrovascular accident in one (14.3%). One patient had no comorbidity. Six patients had an ejection fraction of 60%. One patient was on a ventricular assist device. Four patients (57.1%) had CRF and were on dialysis.

Abdominal pain was present in all patients (100%). Three patients (42.9%) had nausea and vomiting.

**Figure 1:**
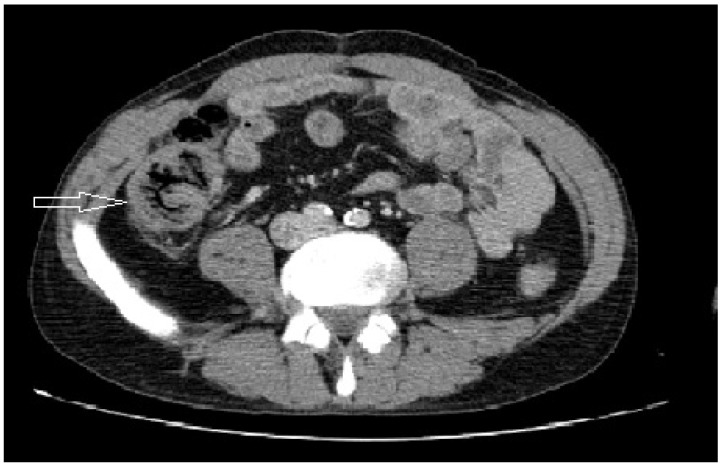
Cecal wall thickening and air in bowel wall in ICN (white hollow arrow).

Computed tomography (CT) and ultrasound (USG) were performed in four and two patients, respectively. CT demonstrated air in the bowel wall in one of the two perforated cases and cecal wall thickening with mesenteric contamination in the other. Two non-perforated patients had wall thickening and mesenteric contamination and no specific CT findings (Figure 1). The USG findings were compatible with acute appendicitis in one of the non-perforated cases and were non-specific with the exception of the presence of intra-abdominal fluid in the other of the non-perforated cases (Table 1).

**Figure 2:**
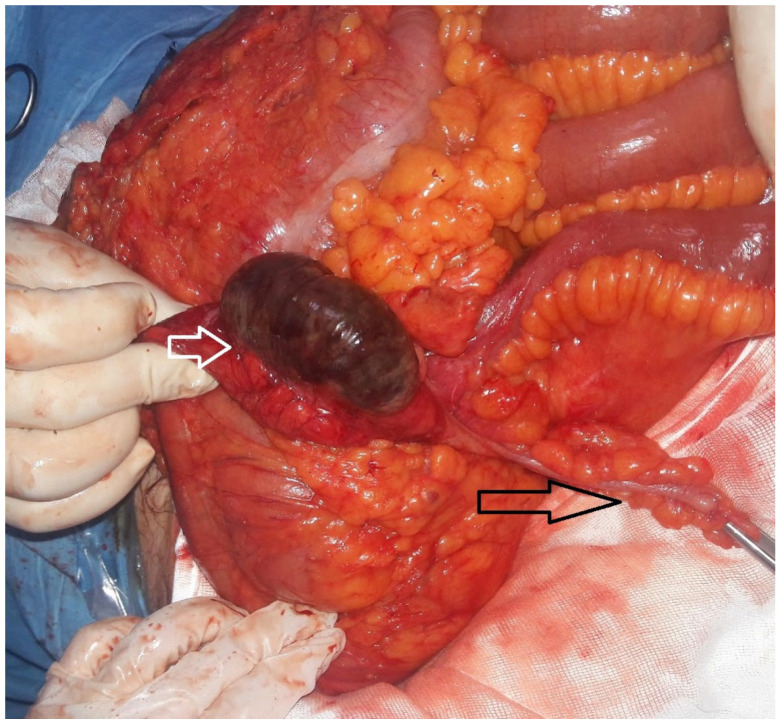
Isolated cecum necrosis (white hollow arrow). The black hollow arrow shows the appendix.

Five patients were operated on with a prediagnosis of acute abdomen (71.4%) and two patients with acute appendicitis (28.5%). Cecal necrosis was observed during surgery in all patients (Figure 2).

The median interval between symptoms and surgery was 1 day (range 1–4 days).

There were two patients with perforation, one female and one male. One patient (33%) with perforation had CRF. The median age of patients in the perforated and non-perforated groups was 61 (range 53–62) and 61.5 (range 36–67) years, respectively. The median WBC count was 14.5 × 10^9^/L (range 9.0–20.8 × 10^9^) and the WBC/neutrophil ratio of patients in the perforated and non-perforated groups was 1.19 and 1.13, respectively.

All patients underwent conventional right hemi-colectomy. No laparoscopic surgery was performed. An ileocolic anastomosis was performed in four patients (57.1%) and a terminal ileostomy was performed in three patients (42.8%).

Perforation due to cecal necrosis occurred in three patients (42.9%). Two of them (28.6%) had signs of purulent peritonitis and underwent resection + terminal ileostomy. One patient underwent resection + ileocolic anastomosis. All patients were discharged from hospital without any complications. Two of the patients with perforation had CRF and in one of them a terminal ileostomy was performed and in the other, an anastomosis was performed without complications.

In the non-perforated cases, end ileostomy was performed in one patient, and anastomosis was performed in three patients. The two patients with CRF in the non-perforated group who underwent anastomosis were re-operated on postoperative days 6 and 3, respectively, because of hematoma formation after hemodialysis in one patient and anastomotic leakage in the other patient. An end ileostomy was performed in the patient with anastomotic leakage, and the hematoma was drained in the other patient whose anastomosis was intact. However, the hematoma-drained patient developed anastomotic dehiscence on postoperative day 3 and underwent a terminal ileostomy. Unfortunately, the patient died of sepsis on postoperative day 16. None of the remaining patients died due to ICN but due to other causes between 4 and 90 months. Only one patient is still alive 60 months after surgery.

The median hospital stay for all patients was 12 (range 4–16) days. The median hospital stay for patients who underwent a terminal ileostomy and patients who underwent anastomosis at the initial surgery was 7 (range 4–13) and 13 (range 11–16) days, respectively. The median hospital stay for patients with and without perforation was 7 (range 4–12) and 13.5 (range 11–16) days, respectively.

## 4. Discussion

As isolated cecal necrosis is rare, case reports are usually found in the literature [1,5,9,10,11,12,13,14,15]. The largest series consisted of 13 and 17 patients, respectively [16,17]. These studies reported a mean age of 68 (51–84) and 56 (22–85) years, respectively [16,17]. In our review of the literature, there was a wide age range of 38–91 years [1,2,3,4,5,6,7,8,9,10,11,12,13,14,15,16,17]. The age range of our patients was 36–67 years. The youngest patient in our study had CRF and was undergoing hemodialysis. Thus, this study showed that ICN may be seen in young patients with comorbidities.

There are two types of isolated cecal necrosis: Type 1 occurs spontaneously and there is no identified cause, although there may be some concomitant comorbidities such as congestive heart failure, ischemic heart disease, hypertension, diabetes mellitus, or a history of drug use such as glypressin, prednisolone, digoxin, etc., that may precipitate the event. Here, the mechanism is thought to be mesenteric arterial vasoconstriction as a response to hypotension. Similarly, digoxin causes ICN by the same vasoconstrictive effect [4].

Type 2 necrosis occurs secondary to decreased mesenteric perfusion in situations such as decreased cardiac output resulting in systemic hypotension, cardiac surgery, cardiopulmonary bypass, or hypotension in hemodialysis patients [4,14,15].

Since the cecum has the largest diameter and is a “watershed” in terms of blood supply, it has been proposed to be more susceptible to ischemia, especially in the absence of a vascular arcade between the ileal and colic arteries [13].

The most common symptoms reported in ICN are RLQ pain and nausea/vomiting, respectively [1,2,3,4,5,6,7,8,9,10,11,12,13,14,15,16,17]. These symptoms were also reported in our cohort. However, other symptoms reported in the literature such as rectal hemorrhage, diarrhea, and abdominal distension were not seen in our patients [2,3,4,5,6,7,8,10,11,12,13,14,15,16,17].

In particular, hemodialysis patients are at higher risk for ICN due to arterial vascular disease and the risk of hypotension during hemodialysis [2,3,16]. CRF was the most common comorbid condition and the only fatal case in our series also had CRF.

In our study, six patients had comorbidities. Four patients had CRF and were treated with hemodialysis, and one patient had an artificial heart. DM, HT, and CAD were other comorbidities. Thus, both type 1 and type 2 factors were present in our patients [14]. Our findings correlated with the literature regarding the risk of hemodialysis patients. One 59-year-old patient had no known comorbidities. Hunter et al. also reported a 74-year-old ICN patient without comorbidities in their series [14]. Thus, ICN may occur in the elderly without known comorbidities.

The diagnosis of isolated cecal necrosis is based on suspicion, especially in elderly patients with comorbidities. Abdominal pain in the RLQ is the main symptom of ICN, which may interfere with other pathologies presenting with pain in this area. Nausea and vomiting, bloating, diarrhea, and rectal bleeding may accompany the pain (Table 3).

ICN should be considered in the differential diagnosis of Crohn’s disease, malignancy, phlebitis, and acute appendicitis, especially in elderly patients with comorbidities [4,5,6,15,16]. Two of our patients underwent surgery for a prediagnosis of acute appendicitis, and ICN was observed during surgery. The leukocyte count was reported to be higher than 10,000/µL [4,5]. In our series, the mean leukocyte count was higher than 10,000/µL in non-perforated cases and 15,000/µL in perforated cases, respectively (Table 2). In one study, although the leukocyte count was reported to be higher than 15,000/µL, there is no information on whether the perforation was present or not [2]. In the largest case series by Gundes et al., none of the patients had a “serious” perforation and the abdomen was clean, although half of their cases had a leukocyte count greater than 15,000/µL [16]. In the studies we reviewed, the leukocyte count ranged from 8100–29,000 [1,2,3,4,5,6,7,8,9,10,11,12,13,14,15,16,17]. However, data on the presence of perforation were unclear in most of these studies.

In our opinion, a leukocyte count higher than 15,000/µL is a risk for perforation, but more data are needed.

US imaging showed no specific findings in most studies, including our study [2,5,6,9,16]. It showed free fluid or findings. Therefore, the US may not be a useful diagnostic tool in the diagnosis of ICN.

Contrast-enhanced computed tomography (CT) appears to be a more helpful diagnostic tool. It can show isolated circumferential wall thickening, pneumatosis coli, or perforation [1,2,6]. However, the diagnosis may still be unclear. Then, diagnostic laparoscopy is a good method for definitive diagnosis [1,2,9,14,17]. The laparoscopic approach can allow either to decide the type of incision to be made if converted or to perform the operation laparoscopically [1,17].

Colonoscopy has been used as a diagnostic tool in some cases, especially when the cecal tumor is suspected instead of ICN [7,8,10]. Some studies reported that some cases of ICN with cecal mass responded to medical treatment [8,10]. However, of course, this modality cannot be used in cases with diffuse peritonitis.

In our study, CT scan and ultrasonography (USG) were the imaging tools and diagnostic laparoscopy or colonoscopy were not performed. All patients underwent conventional surgery. USG did not reveal anything specific for ICN. However, CT showed suspicious findings for isolated cecal necrosis in three out of four patients (Table 1). Thus, intravenous contrast-enhanced CT seems to be more useful in the diagnosis of ICN.

It was reported that right hemi-colectomy with anastomosis was performed in patients with peritonitis, and cecal resection with ileocolostomy was performed in a patient with purulent peritonitis without complications or mortality [2]. It has also been recommended to avoid anastomosis in isolated cecal necrosis if necrosis is present [3]. In our study, anastomosis was performed in four patients in the non-perforation group and only one patient with CRF died who had hematoma development after hemodialysis. Although the hematoma was surgically drained, the anastomosis became detached, and a terminal ileostomy was performed. Thus, anastomosis may be an option for both non-perforated and perforated isolated cecal necrosis in the absence of purulent peritonitis. However, caution should be exercised when deciding between anastomosis and terminal ileostomy, especially in hemodialysis patients with CRF or those on antiplatelet drugs due to bleeding tendencies. Furthermore, hypotension and hypoperfusion may jeopardize the anastomosis in the postoperative period. In our study, anastomotic dehiscence was observed in a patient with HT and CAD and an end ileostomy was performed and he was discharged uneventfully.

As the three patients with end ileostomy had a short and uneventful hospital stay, and there were morbidities in two of the patients with anastomosis, one of which was fatal due to anastomotic leakage, it may be more appropriate to perform ileostomy instead of anastomosis in patients with comorbidities, especially those who are on hemodialysis and with hypotension tendency.

Some studies have reported success with medical treatment in selected patients [8,10]. However, as they pointed out, these situations seem to be chronic occlusive and non-occlusive conditions rather than acute ones [8,10].

Most of the studies in the literature did not report any perioperative mortality [1,2,5,6,7,8,9,10,11,12,13,14,15]. However, Gundes, Cakar, Schuler, and Atici reported a perioperative mortality rate of 38, 83, 20, and 35%, respectively [3,4,16].

Gundes et al. stated the prognostic factors as the time from onset of the symptoms to surgery, age, and the number of associated diseases [16]. Cakar et al. suggested that delayed diagnosis is the most important prognostic factor in mortality and ostomy should be preferred instead of making anastomosis after resection if necrosis is present [3].

Karabay et al. stated that morbidity and mortality can be prevented by early surgical treatment [9]. We had one mortality out of seven (14%). The patient was young and had chronic renal insufficiency. Therefore, in any patient with comorbidity, regardless of age, RLQ abdominal pain should bring ICN to mind in the differential diagnosis.

According to our results, although ICN is a life-threatening disease that may occur at a younger age, it does not always have a poor prognosis, especially if early diagnosis and intervention are made and precautions are taken for comorbidities. In the postoperative period, the factors leading to hypotension, fluid deficit, and decrease in hemoglobin level may lead to the progression of ischemia to necrosis or anastomotic dehiscence, although opinions on whether or not progression of necrosis can occur in the residual colon after surgery are controversial [3].

On the contrary, excessive fluid overload in the postoperative period might also increase the risk of anastomotic leakage due to edema in the anastomosis. This situation suggests that comorbidities may increase morbidity and mortality in the postoperative period, regardless of age [18].

The use of McBurney’s incision, especially in patients with a prediagnosis of acute appendicitis, may cause additional problems, such as the need for an additional incision resulting in anterior abdominal wall defects and restriction of the stoma area if a stoma opening is indicated. Therefore, if ICN is suspected, it is appropriate to begin with a diagnostic laparoscopy to make a definitive diagnosis and determine the optimal incision [8,17]. Unfortunately, our patients underwent laparotomy. A midline incision was preferred in five patients undergoing surgery for acute abdomen. In two patients with a prediagnosis of acute appendicitis, the Mc Burney incision was preferred and converted to a midline incision, demonstrating the importance of diagnostic laparoscopy in such cases.

According to our results, ICN should be considered in the differential diagnosis of any patient of any age with comorbidities who presents with abdominal pain in the RLQ region. These patients should undergo contrast-enhanced CT. If the diagnosis is not clear, diagnostic laparoscopy should be performed.

This study had several limitations. First of all, the number of patients was very small and it was from a single center. However, our number of cases is actually not small, considering the rarity of the pathology and review of the literature. It might have been better to do a multicenter study to increase the number of patients. Second, due to the small number of patients, we could not perform statistical analysis. In the future, a multicenter study with a larger number of patients may provide statistically comparable results.

In older or even younger patients with comorbidities who present with right lower quadrant pain, ICN should be considered and a contrast-enhanced CT should be ordered. If diagnostic uncertainty exists, a diagnostic laparoscopic procedure would be preferable to avoid unnecessary incisions.

If ICN is the diagnosis, an ileocecal resection or a right hemi-colectomy can be performed, and especially in patients on hemodialysis and anticoagulants, an end ileostomy may be a good option instead of an anastomosis.

## 5. Conclusions

Although it is more common in older patients with comorbidities, ICN can also occur in the young. With early diagnosis and surgery, it may have a better prognosis. In patients with comorbidities who present with sudden pain in the RLQ, it should be suspected. Contrast-enhanced CT is a good imaging technique for diagnosing ICN. Laparoscopy is a good option in patients with comorbidities, even if acute appendicitis is the prediagnosis, to establish the diagnosis and determine the type of incision in the case of ICN. A leukocytosis greater than 15,000 may be a sign of perforation. In the presence of purulent peritonitis or in patients with a tendency to hypotension, such as dialysis patients and patients with coronary artery disease, an ileostomy should be preferred. Perioperative fluid administration should be strictly controlled in all patients, but especially in those with anastomosis.

## Figures and Tables

**Table 1:** Patient demographics.

**The number of patients**	7
**Age (median and range)**	61 (36–67)
**M/F ratio**	5/2
**Symptoms**	
Pain	7/7
Nausea and vomiting	3/7
**Symptom duration (days) (median and range)**	1 (1–4)
**Prediagnosis**	
Acute abdomen	5/7
Acute appendicitis	2/7
**Chronic Disease**	6/7
CRF	4/7
DM	2/7
CAD	2/7
HT	2/7
ADPKD	1/7
CVA	1/7
TAH	1/7
None	1/7
**Ejection fraction (%)** (6/7)	60
**Dialysis treatment**	4/7
AVF	4/7
**USG**	2/7
**USG findings**	Intra-abdominal free fluid
	Findings consistent with acute appendicitis
**CT**	4/7
**CT findings**	
Non-specific	2/7
Air in the bowel wall	1/7
Increase in thickness of the cecum wall	2/7
**Perforation**	3/7
**The interval between the beginning of the symptoms and surgery (Day)** **(median and range)**	1 (1–4)
**Incision**	
Midline laparotomy	5/7
McBurney	2/7
**Surgical procedure**	
Right hemi-colectomy + anastomosis	4/7
Right hemi-colectomy + end ileostomy	5/7
Hematoma drainage	1/7
Conversion to end ileostomy	2/7
**Mortality**	1/7
**Hospital stay (day) (median and range)**	12 (4–16)
**30-day mortality**	1/7

CRF: chronic renal failure; DM: diabetes mellitus; CAD: coronary artery disease; HT: hypertension; ADPKD: Autosomal Polycystic Kidney Disease; CVA: cerebrovascular accident; TAH: The Artificial Heart; AVF: arteriovenous fistula; USG: ultrasonography; CT: computed tomography.

**Table 2:** Comparison of the patients with and without perforation.

		Perforation	Non-Perforation
**Gender**	M	1	4
	F	2	0
**Age (median)**		61	61.5
**Abdominal Pain**		3	4
**Nausea–Vomiting**		1	2
**CRF**		2	2
**CAD**		1	1
**DM**		1	1
**HT**		0	2
**CVA**		1	0
**TAH**		1	0
**ADPKD**		0	1
**Surgery**	Ileostomy	2	1
	Anastomosis	1	3
**2nd Operation**	Ileostomy	-	1
**3rd Operation**	Ileostomy	-	1
**Mortality**		0	1
**WBC (×10^9^/L)**		16.783	12.8525
**NEUT (mcL)**		14.61	11.4975
**WBC/NEUT**		1.187	1.077
**Hb (g/dL)**		11.53	12.9
**HCT (%)**		37.2	39.875
**PLT (/mcL)**		324,000	212,000

ADPKD: Autosomal Polycystic Kidney Disease; CAD: coronary artery disease; CRF: chronic renal failure; DM: diabetes mellitus; CVA: cerebrovascular accident; g/dL: gram per deciliter; Hb: hemoglobin; HCT: hematocrit; HT: hypertension; L: liter; M: male; mcL: microliter; F: female; TAH: The Artificial Heart; WBC: white blood cell; PLT: platelet.

**Table 3:** Some studies on ICN.

Study/ (Peri-Operative Mortality)	Number (M/F)	Age (Years)	Symptom	Symptom Duration (Day)	WBC Count (/mm^3^)	US	CT Imaging	Colonoscopy	Comorbidity	Preliminary Diagnosis	Incision	Procedure
**Rist 1984** **(0)**	3 (M)	79 (59–84)	3xPain, 2xNausea, Vomiting	-	16.000 (12.600–18.100)	-	-	-	3xCHF, 2xCVD (Digoxin) 1xCVA	2xAcute abdomen 2xAcute appendicitis	-	2xRight hemicolectomy 1 Cecectomy
**Schuler 2000** **(1/5)**	5 (1/4)	84 (57–91)	5xPain, 1xNausea/ Vomiting, 1x Diarrhea	1 (0.3–3)	17.000 (12.000–20.600)	-	-	-	2xHT 1xDM 1XCABG 1xCAD 1xCHF	Acute appendicitis Acute abdomen Cecum cancer	-	4xRight hemicolectomy +anastomosis 1xRight hemicolectomy +end ileostomy
**Ruiz-Tovar 2008** **(0)**	1F	82	Pain	4	8.100	-	Asymmetric thickening of the caecal wall, suggesting a caecal neoplasm			Cecum tumor	Midline laparotomy	Right hemicolectomy +anastomosis
**Dirican 2009** **(0)**	4 (2/2)	59 (46–68)	4xPain 4xNausea/ Vomiting		20.200 (16.400–23.700)	3xFree intra abdominal fluid	1xNon-specific	-	2xCRF 1xCRF+DM+HT 1xCOPD	Acute appendicitis	1xDiagnostic laparoscopy 3xLaparotomy	3xRight hemicolectomy +anastomosis 1xCecum resection +ileostomy
**Gundes 2013** **(5/13)**	13 (8/5)	68 (51–84)	13xPain 8xDistention, 8xVomiting	3 (1–7)	15.200 (8.700–29.000)	5xFluid in the right lower quadrant and contamination in the fatty planes 3x Normal	2xThickening and inflammation in the cecal wall	-	5xCRF 3xHT 2xAF 2xDM 1xCOPD 1xFMF 1xCAD 1xCVA	Acute appendicitis	-	10xRight hemicolectomy 2xRight hemicolectomy +end ileostomy 1xCecal resection
**Hunter 2013** **(0)**	1F	74	Pain Nausea	0.25	12.030	-	-		HT Diverticulosis	-	Diagnostic laparoscopy +Midline laparotomy	Partial cecum resection
**Çakar 2014** **(5/6)**	6 (3/3)	60.3 (38–85)	Pain Vomiting	-	-		1xAir-fluid level (out of 4)	-	4xCRF 4xDM 4xHT 1xAF+CAB 1xAortabifemo- ral graft	Acute appendicitis	1xDiagnostic laparoscopy Laparatomy	2xRight hemicolectomy +end ileostomy + mucous fistula 2xRight hemicolectomy +anastomosis 1xCecal resection +anastomosis+ 1xCecal resection +end ileostomy + mucous fistula 3xReoperation 2xEnd ileostomy+mucous fistula 1xRight hemicolectomy +end ileostomy +mucous fistula
**Shahverdi 2017** **(0)**	1 (F)	62	Pain Nausea Vomiting	-	9.700	Dilated bowels with abundant gas	A minor fluid collection in right lower quadrant	-	DM, HT, CAD, CVA, CABG Behçet’s disease	Acute abdomen	Midline	Right hemicolectomy + anastomosis
**Karabay 2018** **(0)**	1 (F)	68	Pain	7	15.390	Non specific	Linear density at the back of the cecum and a tubular structure extending to the liver	-	DM, HT	Acute appendicits	Laparoscopy +open surgery	Partial cecum resection +anastomosis
**Kohga 2018** **(0)**	1 (M)	59	Pain	0.2	8.700	-	A dilated cecum surrounded by free air	-	AF, digoxin	Cecum perforation	Diagnostic laparosocpy	1xLaparoscopic assisted ileocecal resection +anastomosis 1xIleostomy
**Chan 2018** **(0)**	1 (F)		Pain Nausea Vomiting/ and Diarrhea.		10.000	-	Focal cecum ischemia and chronic SMA stenosis	Cecal ulcer and ischemic mucosa	AF, HT, CRF	Cecum ischemia	-	Medical treatment
**Eyvaz 2020** **(0)**	1 (F)	76	Pain, Nausea	0.5	16.200	-	Thickening on the cecum wall	-	TBC Thyroidectomy HT	Acute abdomen, Acute appendicitis	Midline	Ileocecal resection +anastomosis
**Kardoun 2021** **(0)**	2 (F)	72 (66–78)	2xPain 1xNausea/ Vomiting	-	12.850 (11.600–14.100)	-	1xDilated cecum with mural thickening, edema, and intramural gas (pneumatosis), portal venous gas and mesenteric gas while (appendix normal) 1xCecum surrounded by free air, (appendix normal)		2xCRF 2xHT 1xCAD 1xDyslipidemia 2xDM 1xAF (Digoxin)	Cecum ischemia	Midline laparotomy	1xCecum resection +anastomosis 1xIleocecal resection + a double-barrel ileocolostomy
**Atıcı 2022** **(6)**	17 (9/8)	56 (22–85)	17xPain 17xNausea	1			17xPericecal inflammation and cecal wall thickening		4xCRF 5xCAD 8xHT 3xCHF 5xArrhytmia 4xDM 3xCOPD 1xChronic pancreatitis 1xLung cancer 1xIliac artery stent 1x Aplastic anemia		2xDiagnostic laparoscopy 17xMidline	14xRight hemicolectomy +anastomosis 2xRight hemicolectomy +Mikulicz ileocolostomy 1xPartial cecum resection +Mikulicz ileocolostomy
**Suleimanov 2022** **(0)**	1 (M)	42	Pain Nausea/ Vomiting	3	16.000	Unremarkable		-	-	Acute appendicitis	McBurney +extension	Ileocecal resection +anastomosis
**Janike 2023** **(0)**	1F	77	Pain Nausea Vomiting Melena Wigth loss	Several days	13.000	-	Colonic mass	Cecal ischemic mass	HT Hyperlipidemia Obesity	ICN	Laparotomy	Right hemicolectomy + anastomosis
**Liao 2025** **(0)**	11 (4/7)	72 (43–87)	6xPain 5xGI bleeding 3xDiarrhea 4xNausea/ Vomiting 2xAsymptomatic (screening colonoscopy)	-	-	-	8xColonic mass (out of 9)m (pathology ischemia)	9x Colonic mass (out of 9)	7xHT 5xCVD 4xHyper-lipidemia 2xCOPD 2xDM 1xCRF	ICN	5x Laparotomy	6xMedical Treatment 5xRight hemicolectomy

## Data Availability

The data is unavailable to the public because of ethical restrictions but can be asked from the corresponding author.

## Abbreviations

The following abbreviations are used in this manuscript:

ICN	Isolated cecum necrosis
ADPKD	Autosomal Polycystic Kidney Disease
AVF	Arteriovenous fistula
CAD	Coronary artery disease
CRF	Chronic renal failure
DM	Diabetes mellitus
CVA	Cerebrovascular accident
CT	Computed tomography
F	Female
g/dL	Gram per deciliter
Hb	Hemoglobin
HCT	Hematocrit
HT	Hypertension
L	Liter
M	Male
mcL	Microliter
PLT	Platelet
RLQ	Right lower quadrant pain
TAH	The Artificial Heart
USG	Ultrasonography
WBC	White blood
